# *Saccharopolyspora erythraea’s* genome is organised in high-order transcriptional regions mediated by targeted degradation at the metabolic switch

**DOI:** 10.1186/1471-2164-14-15

**Published:** 2013-01-16

**Authors:** Esteban Marcellin, Tim R Mercer, Cuauhtemoc Licona-Cassani, Robin W Palfreyman, Marcel E Dinger, Jennifer A Steen, John S Mattick, Lars K Nielsen

**Affiliations:** 1 Australian Institute for Bioengineering and Nanotechnology (AIBN), The University of Queensland, Brisbane, Qld, 4072, Australia; 2 Institute for Molecular Bioscience (IMB), The University of Queensland, Brisbane, QLD, 4067, Australia

**Keywords:** RNA-sequencing, actinobacteria, Saccharopolyspora erythraea, Erythromycin, Metabolic switch

## Abstract

**Background:**

Actinobacteria form a major bacterial phylum that includes numerous human pathogens. Actinobacteria are primary contributors to carbon cycling and also represent a primary source of industrial high value products such as antibiotics and biopesticides. Consistent with other members of the actinobacterial phylum, *Saccharopolyspora erythraea* undergo a transitional switch. This switch is characterized by numerous metabolic and morphological changes.

**Results:**

We performed RNA sequencing to analyze the transcriptional changes that occur during growth of *Saccharopolyspora erythraea* in batch culture. By sequencing RNA across the fermentation time course, at a mean coverage of 4000X, we found the vast majority of genes to be prominently expressed, showing that we attained close to saturating sequencing coverage of the transcriptome. During the metabolic switch, global changes in gene expression influence the metabolic machinery of *Saccharopolyspora erythraea,* resetting an entirely novel gene expression program. After the switch, global changes include the broad repression of half the genes regulated by complex transcriptional mechanisms. Paralogous transposon clusters, delineate these transcriptional programs. The new transcriptional program is orchestrated by a bottleneck event during which mRNA levels are severely restricted by targeted mRNA degradation.

**Conclusions:**

Our results, which attained close to saturating sequencing coverage of the transcriptome, revealed unanticipated transcriptional complexity with almost one third of transcriptional content originating from un-annotated sequences. We showed that the metabolic switch is a sophisticated mechanism of transcriptional regulation capable of resetting and re-synchronizing gene expression programs at extraordinary speed and scale.

## Background

Actinobacteria represent one of the most dominant and distinct bacterial phylum. The evolutionary divergence of actinobacteria is an ancient event, reflected in their unique biology. The majority of actinobacteria are found in soil, where they play a vital role in carbon cycling. In human medicine, they comprise a large number of pathogens and provide a major source of antibiotics [1]. They have a large, high GC content genome that equips them for growth in hostile, nutrient poor environments. They are also able to synthesise secondary metabolites that have important commercial and medical significance [2].

*Saccharopolyspora erythraea* (*S.erythraea*) is a model gram-positive filamentous actinomycete that has served for the study of antibiotic production for several decades. The bacteria produces erythromycin A, the first macrolide antibiotic to be discovered, and the backbone for numerous modern antibiotics [3]. *S.erythraea* exhibits a distinct and complex lifecycle, in which an initial growth phase is followed by a transition period, known as the metabolic switch, followed by a secondary growth phase. Like in *Streptomyces coelicolor* (*S.coelicolor*), the switch in *S.erythraea* is followed by morphological changes that coincide with potential cell death and the transcription of secondary metabolite gene clusters [4]. A detailed study of transcriptional activity during this transitional period is of major importance to understand the complex, yet little understood, life cycle of this sophisticated bacterial phylum. Studies have profiled actinomycete transcriptomes using microarrays [5,6]. In fact all annotated coding sequences have been profiled using Affymetrix arrays in *S.coelicolor*[7]. However, using long probes which are less likely to produce false negatives has proved challenging due to the high GC content of actinobacterial genomes. The recent advent of RNA sequencing provides a new opportunity to profile the global transcriptome of high GC content microorganisms.

Here, we present a detailed transcriptional description of *S.erythraea* life cycle from in-depth RNA sequencing. Our study shows that the major morphological changes observed at the metabolic switch are accompanied by an extreme transcriptional phenomenon, with the repression of large regions of the genome. In addition, we characterise a restrictive bottleneck event mediated by targeted RNase activity that results in a wholesale and rapid global transcriptional rearrangement event.

## Results

### RNA sequencing shows pervasive transcription of the genome

Like most actinobacteria, *S.erythraea* has a 71.1% GC content genome which prevented us designing and performing microarray experiments that contained all ORFs (Additional file 1: Figure S1). After performing microarray experiments in a control sample, we employed RNA sequencing to profile the whole transcriptome. We first assessed the ability of two alternative RNA sequencing approaches, duplex-specific thermostable normalisation (DSN) [8] and MicrobExpress depletion [9], to deplete rRNA in GC-rich transcriptomes. Enriched mRNA samples were compared to untreated matched reference controls (Additional file 1: Figure S1B,G,H). We did not observe any systematic divergence in nucleotide composition of sequenced reads between treated and untreated libraries (*r*^*2*^ = 0.01, Additional file 1: Figure S1B-H). We observed a high correlation between gene abundance estimates by independent validation by qRT-PCR and the expression of low GC content genes represented in the microarray (Additional file 1: Figure S1I,J), thereby confirming the fidelity of both strategies to profile GC rich transcriptomes.

**Figure 1:**
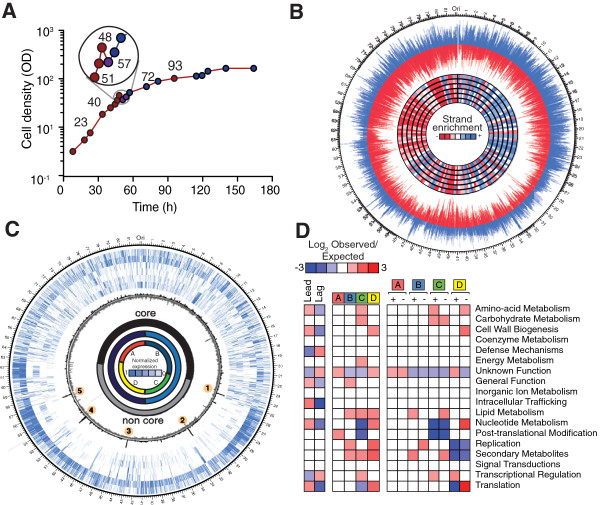
**Transcriptional topography. (A)** Profile of *S.erythraea* fermentation cycle according cell density. Time points sampled for RNA-seq across primary (red), transition (purple) and secondary (blue) metabolic phases are indicated. **(B)** Genome plot showing leading and lagging strand transcriptional bias. Outer circle shows histogram of transcription from sense (blue) and antisense (red) during the metabolic switch (51 hours). Inner-circle shows binned strand preference across sampled time points from the duplicate fermentation sequenced strand specifically after MicrobExpress enrichment. **(C)** Genome plot showing core to non-core transcriptional macro-regions after normalization. Outer circle (blue) normalised expression across sampled time points (outer circle represents the first time point (for clarity, the metabolic switch time point was excluded from the plot). Inner circle indicates binned enrichments for core/non-core regions, with switching of region enrichment observed at the switch. Four macro-regions annotated from superimposition of leading/lagging and core/noncore regions are indicated (red, blue, green and yellow). Numbered dots (1–5) indicate long inverted repeats. **(D)** Grid-plot indicates the enrichment for alternative functional gene categories in the four macro-regions in strand specific and core/non-core regions (only p < 0.05 indicated). A,B, C and D are the transcriptional macro regions observed in Figure 1C (red, blue, green and yellow).

Prior to harvesting RNA, several fermentations were performed to carefully characterize the metabolic switch in bioreactors. We determined that RNA harvested at seven time-points, (three points immediately before, one during and three following the metabolic switch) would accurately describe the entire genetic program (Figure 1A ). We prepared unstranded RNA libraries [10] and sequenced 363 million reads for a total of 32.7 Gb and a mean ~4,000 fold coverage of the genome (Additional file 2: Table S1, Additional file 2: Table S2). A replicate fermentation was sampled across the metabolic switch (Additional file 1: Figure S2C). The replicate samples were used to prepare strand-specific libraries that were sequenced to produce 86.1 million alignable reads and an additional 7.5 Gb of sequencing (Additional file 2: Table S1).

Our comprehensive sequencing showed the *S.erythraea* genome to be prevalently transcribed, with at least 85.8% of genome sequence from either strand being represented (98.7% of the genome without considering strand). This is similar to recent studies in yeast and other eukaryotes that show pervasive transcription of complex genomes that express intergenic and noncoding RNA [11,12]. Furthermore, given less than 0.3% of expressed nucleotides were represented singly suggests that close to saturating sequencing coverage of the transcriptome was attained, thereby permitting us to detect rare and transient transcriptional events.

### The transcription of genomic macro-regions alternates at the switch

We next applied RNA sequencing to resolve large-scale topological features of the *S.erythraea* transcriptome. Our sequencing clearly illustrates the strand-specific enrichment for transcription from the leading strand, consistent with co-directional transcription of essential and highly expressed genes with replication [13] (Figure 1B). Like other actinomycetes, the genome is divided into two large regions, with a core region thought to contain the majority of genes essential for survival, and a non-core region enriched for genes for conditionally adaptive functions [2,14]. Alignment of sequenced reads across these regions allowed the resolution of genome-scale, contiguous transcriptional regions associated with these core/non-core regions (Figure 1C). These macro-regions undergo alternative enrichment across the switch, with marked repression of the non-core transcriptional loci following the switch (Figure 1C).

**Figure 2:**
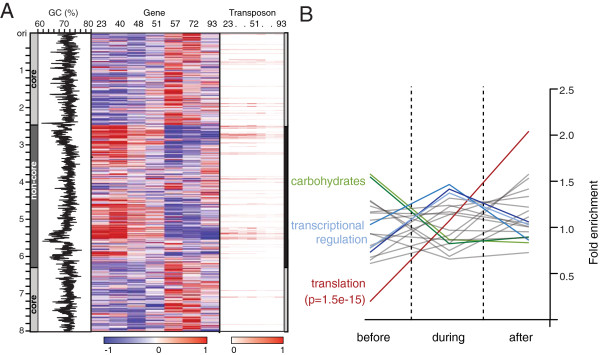
**Genome architecture.** (**A**) Heatmap indicating the switching of core (grey) and noncore (dark grey) transcriptional macro-regions during fermentation as follows: black histogram (left panel) shows % of GC with low GC demarcating region border; gene expression (left/middle panel (red and Blue); centred to mean) and transposon expression (red right panel). Together gene expression and transposons show a structured genomic organisation relative to macro-region architecture. (**B**) Two alternative genetic programs are demarcated at the switch by a bottleneck event that is readily apparent in transcriptomic data. Significant enrichment for functional gene categories at different stages of the growth cycle is indicated.

The borders of the core/non-core macro regions are delineated by transposable sequences (Figure 2 and Additional file 1: Figure S2A) [2]. Given the capacity for RNA sequencing to resolve the expression of repetitive sequences, we were able to detect a notably strong and specific induction (p = 6.5x10^-7^) of paralogous transposon clusters at these region borders, coincident with the shifting of non-core to core transcriptional regions. These borders orientate the transcriptional regions perpendicular to the strand-specific enrichment for leading/lagging strands (Figure 1B). When superimposed, these regions dissect the genome into four distinct quarters; with each region containing distinctly co-expressed and functionally related gene families (Figure 1C,D). Collectively this demonstrates a broad regulatory context provided by contiguous transcriptional macro-regions for the functional organisation and alternate expression of genes across the metabolic switch.

### The metabolic switch defines a distinct gene expression program

The high temporal resolution and sequencing depth with which we profiled the growth cycle and metabolic switch using RNA-seq (with >4000 fold genome coverage), revealed considerable complexity in gene expression profiles. Although we detected transcription from all genes, for clarity we restricted our analysis to prominently expressed genes (top 50% of the total most prominently expressed genes). Shifts to gene expression invoked at the switch were so broad as to affect 60.5% of the genes that were profiled (>2-fold change). To delineate cogent trends within this complexity, we identified major gene groups that exhibited a normalised enrichment at exponential, transitional or stationary phases across the *S.erythraea* life cycle (Additional file 1: Figure S3A-C, S4A-C). A comparative analysis delineated two distinct gene expression programs with diverging functional trends before and following the switch (Additional file 2: Table S2). For example, the greater number of genes expressed prior to the switch were enriched for programs associated with central carbon metabolism, such as those involved in carbohydrate synthesis (p = 1.75x10^-16^), cell wall biogenesis (p = 0.043), energy production (p = 6.80x10^-4^) and lipid metabolism (p = 0.011). In contrast, genes associated with secretion and transport (p = 0.037), posttranslational modification (p = 8.98x10^-6^) and ribosomal structure and biogenesis (p = 1.12x10^-16^) were enriched after the switch. Similarly, the erythromycin gene cluster was enriched after the metabolic switch (Additional file 1: Figure S3). During the metabolic switch the greatest enrichment was for genes associated with signal transduction (p = 0.0011) and transcription regulation (p = 1.94x10^-9^). These alternate programs were reciprocally enriched for core/non-core and leading/lagging strand organisation (Figure 1D), reflecting the overarching context these macro-regions provide for gene expression. Interestingly we found similar patterns of up or down regulation to previously reported homologous genes in closely related microorganisms [7,15], suggesting that the transcriptional phenomenon described here comprise a characteristic event that punctuates and defines gene expression in related microorganisms (Additional file 1: Figure S8).

### RNA sequencing facilitates operon annotation

By the contiguous transcription across shared and co-expressed genes, we looked at the predicted organization of the 1,134 previously predicted operons that encompass 4,919 genes [16]. Our *de novo* assembly using Oases[17], from the strand specific RNA sequencing after trimming all reads to 70 bases and assembling them using a 45 bases k-mer, resulted in 9,170 contigs. We found several of these contigs aligned with previously predicted operons (Additional file 2: Table S3). Moreover, using the strand specific sequencing, we found 2.9% of total transcription to be antisense to these annotations, with an enrichment of antisense transcription at the 3’ end of the genes. Lastly, we employed a range of metrics, including presence, size and structure to annotate novel independent ncRNAs within intergenic regions. By Combining small RNA sequencing and long RNA-seq data, we identified 190 putative ncRNA from transcribed sections in intergenic genomic regions (Additional file 2: Table S7 and ncRNA track in the genome browser). The novel ncRNAs display distinct CPC scores [18], dynamic transcriptional pattern and 14 of them displayed a distinct ncRNA secondary structure (Additional file 1: Figure S7; Additional file 2: Table S4, S7) [18].

### RNA degradation mediates a transcriptional bottleneck event

**Figure 3:**
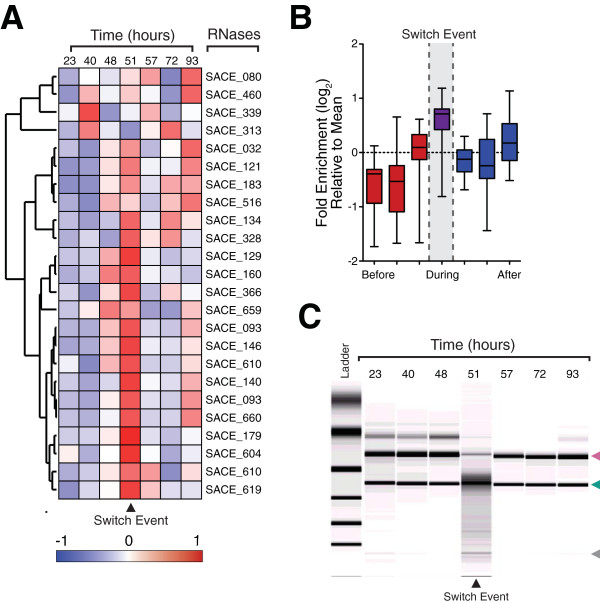
**Targeted degradation of *S.erythraea *transcriptome.** (**A**) Heat map summarising relative expression profile of annotated ribonucleases. Ribonuclease expression profile across the growth cycle shows a collective induction at the switch coinciding with RNA degradation observed at the switch. **(B)** Box-whisker plot (Tukey distribution, n = 24) summarising relative fold enrichment of ribonuclease expression across the metabolic switch. **(C)** Bioanalyzer tracer showing RNA quality sampled across the *S.erythraea* fermentation cycle. RNA shows smeared RNA degradation at switch (arrow) correlating with high RNAse activity.

The two alternative genetic programs are demarcated at the switch by a bottleneck event that is readily apparent in the transcriptome, with up to a 2.14-fold suppression in gene counts for genes associated with translation (Figure 2B). This bottleneck event, happening at the metabolic switch, is aptly illustrated in the suppression of essential components to the transcriptional, translation and energy production machinery (Figure 2B, Additional file 1: Figure S4A-F). This group of genes persisting at the switch was enriched for transcriptional regulators (p = 1.94x10^-9^; Additional file 1: Table S5), consistent with the major phenotypic changes that occur during this phase. Furthermore, the wide imbalance between the highest and lowest expressed genes (the top 1% genes contribute 27.8% of total mRNA) suggests the majority of mRNAs to be present at less than one copy per cell, consistent with recent reports of heterogeneity between individual cell transcriptomes [19]. This imbalance narrows during the bottleneck event, suggesting decreased diversity between individual cell transcriptomes. This observation is in accordance with the previously described programmed cell death that occurs at the switch and involves expression of enzymes for cellular dismantling [20]. Notably, we observed that BioAnalyzer traces from two independent fermentations showed degraded profiles at the switch (Figure 3C, S4H). Furthermore, we observe the pronounced expression of ribonucleases and proteases at the inception of the switch (21 out of 24 ribonucleases; p < 0.0001 paired t-test (Figure 3A,B)), including RNase E and RNase III. Given the critical roles ascribed to these ribonucleases in regulating the half-life of developmental mRNAs in related species [21,22], in combination with specific RNA depletion at the switch, we next considered the role of targeted RNA degradation in the global rearrangement of the transcriptome.

### Degradation targets specific mRNA classes

Our analysis of RNA integrity across the *S.erythraea* life cycle revealed extensive RNA degradation coincident with the metabolic switch (Figure 3C and Additional file 1: Figures S4H). Given that samples were treated with RNA-later and that we obtained high RIN numbers (except for the sample corresponding to the metabolic switch), it was unlikely that degradation could occurred post-extraction. Although the Qiagen columns have less binding affinity for RNAs smaller than 200 bp, it does not completely remove sRNAs [23]. Thus, to provide an insight into the degradation pattern, we isolated and sequenced these degraded RNA fractions. We firstly isolated fragments corresponding to 20-50nt in size, thereby depleting the preparation of small regulatory RNAs that were greater than 50nt in length [24]. We also reasoned that, unlike the 5’ monophosphate termini of a fragmented transcript, primary RNA transcripts with a 5’ triphosphate would be refractory to library preparation and sequencing (Additional file 1: Figure S5D) [25]. For comparison we also sequenced matching fractions from before (40 hours) and after (93 hours) the switch, returning approximately 16 million sequenced small RNAs (Additional file 1: Figure S5C). We next omitted any reads that did not align in sense to transcribed regions from matched RNA sequencing libraries, reasoning they may reflect *bona-fide* small RNAs. Alignment of the remaining small RNA fragments exhibit no apparent size or nucleotide enrichment to annotated coding genes previously resolved.

**Figure 4:**
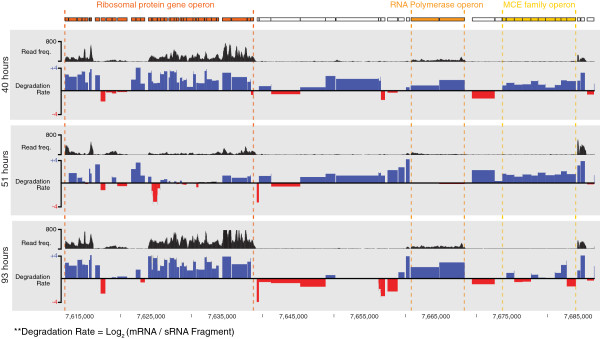
**Genome browser view of mRNA degradation rate.** Genome browser view showing the degradation rate of numerous gene clusters as measured by comparing the representation of genes in full-length to degraded fragment RNA libraries (Log2 (mRNA\sRNA). Full length read frequency (black histogram) also indicated. Differential slower (blue) or faster (red) relative degradation is observed between gene operons across different time points (40, 51 and 93 hours). For example, RNA Polymerase operon (orange) undergoes accelerated decay, as indicated by lower degradation rate, during the metabolic switch.

We next identified those mRNAs subject to targeted degradation and turnover across the switch by comparing the ratio of full-length and fragmented reads that align to each transcript. Although we observed broadly accelerated mRNA degradation at the switch (Additional file 1: Figure S6A), we noted this degradation to be relatively slower or faster for specific genes and functional classes and to often occur in concert with our previously described gene expression profiles. For example, consistent with their enrichment at the switch, we found that while transcripts encoding transcription factors generally exhibit higher stability (2.1-fold, p = 4.5x10^-12^, Additional file 1: Figure S6), some specific classes, such as XRE transcription factors, known to regulate developmental life cycle and secondary metabolism [26], undergo a cohesive and accelerated degradation (Additional file 1: Figure S6F). This observation establishes that the degradation can be targeted to specific mRNA classes, thereby contributing to the stage-specific enrichments observed across the *S.erythraea* growth cycle. Gene ontology analysis of *S.erythraea* genes most intensively targeted for destruction at the metabolic switch returned a large enrichment for transcripts associated with the translational process, including ribosomal genesis (4.6-fold, p = 2.12x10^-5^), ribosomal proteins (1.4-fold, p = 6.3x10^-7^) and other associated translational factors. We detected the targeted degradation of additional functional gene networks, such as energy production and regulation, which undergo suppression at the bottleneck. Collectively, these trends demonstrate the contribution of targeted mRNA degradation to the transcriptional bottleneck and the modulation of genetic programs subsequently deployed (Figure 4). They also anticipate a pivotal and global role for ribonucleases, such as RNase E and III, in regulating the metabolic switch [21,22,27,28].

## Discussion

Our analysis of the *S.erythraea* transcriptome describes a major transcriptional phenomenon occurring during fermentation in bioreactors. A genome-wide transcriptional non-core region, delineated by genes of markedly lower GC content, sparse gene density and inverted repeats, is broadly repressed following the switch. This region organization is present in related microorganisms, including some with linear genomes, such as the terminal regions of the *S.coelicolor* linear chromosome where analogous insertion elements mark the inner boundary of terminal inverted repeats [29]. Similar low GC regions have been reported in *Streptomyces ambofaciens*, where these regions have been associated to genome plasticity and may be used to elucidate mechanisms of evolution [30]. The presence of analogous transposable elements delineating the core/non-core regions is apparent in other related microorganisms anticipating a role in the maintenance and possible regulation of these macro-regions. The alternating expression of these regions, previously observed in *S.erythraea* using microarrays [6], is able to mediate a broad coordinated suite of genetic changes, representing a broad architectural mechanism by which a cell can rapidly modulate large changes in the transcriptome in response to environmental cues [31].

This transcriptional reorganization is complemented by a targeted bottleneck event where mRNAs are degraded. The bottleneck event establishes that the degradation may be targeted to specific mRNA classes, thereby contributing to the stage-specific enrichments following the *S.erythraea* growth cycle. The importance of these degradation processes in global transcriptomic regulation has been recently dissected in yeast [32,33] and individual cases described in *S.coelicolor*, whereby endoribonuclease activity of RNase III has been shown to bind to specific transcript and to regulate antibiotic production [21,34]. Studies have also described the role of (p)ppGpp in regulating mRNA half-life in *S.coelicolor* by inhibiting PNPase activity which preserves mRNA for crucial cellular processes [35-37]. Such widespread degradation may also indicate a role for programmed cell death of a distinct cellular subpopulation, in mediating the changes at the metabolic switch. The new transcriptome observed after the switch may be reflective of the transcriptome of the surviving subpopulation. Collectively, these observations of cellular degradation and targeted mRNA degradation supports the emergence of key, yet little studied, mechanism by which gene expression are regulated.

Our results, which attained close to saturating sequencing coverage of the transcriptome, reveal unanticipated additional transcriptional complexity with almost one third of transcriptional content originating from unannotated sequences. Combining RNA sequencing with sequencing of small RNAs showed the importance of non-coding RNAs in bacterial transcription.

## Conclusions

Like other related microorganisms, *S.erythraea* undergoes a distinct transitional switch that is characterized by growth arrest, morphological changes and the production of secondary metabolites. We show this to be accompanied by a massive transcriptional modulation whose speed and scale, revealed here for the first time by RNA sequencing, represents a significant and novel transcriptional phenomenon. Given similarities shared between the life cycle and genome organization with other microorganisms, we suggest that this transcriptional phenomenon may be a widespread feature across the large critically important bacterial group of industrial microorganisms.

## Methods

### Bacterial strain, growth and fermentation conditions

*S.erythraea* (NRRL2338) was used in this study. All fermentations were conducted in 2L bioreactors (Applikon) in mineral salt medium MM-101 without casaminoacids, as described elsewhere [38,39]. Medium ISP 2 (yeast extract, 4 g/L; malt extract, 10 g/L; Dextrose, 4 g/L; Agar, 20 g/L) was used as solid media for spore germination and seed cultures. Approximately 0.5 mL of glycerol stock was used to inoculate starting cultures in a 500 mL baffled flask with 100 mL of ISP 2 media incubated at 30°C in a rotary shaker (INFORS HT, Bottmingen, Switzerland) at 220 rpm for 30 h. When the seed culture reached an OD_450_ of 2.5 (early stationary phase), a second seed culture (1 L baffled flasks with 150 mL of ISP 2) was inoculated to an initial OD_450_ of 0.3 and incubated under the same culture conditions for 72 h. Cells were then centrifuged at 10,000 rpm at room temperature (Allegra X-15R, Beckman Coulter, USA), washed and resuspended in MM-101 prior to inoculation. Temperature and pH remained constant at 30°C and pH = 7.0 respectively by addition of 20% NaOH or 10.9% HCl. Dissolved oxygen was maintained between 45 and 60% saturation. Oxygen uptake rate (OUR) and CO_2_ production were measured using a DASGIP Off-Gas Analyzer GA4 (DASGIP AG, Jülich, Germany) or a mass spectrometer (Hiden HPR20-QIC B, Warrington, England) attached to the bioreactor condensers. Cells were harvested from two biological replicates at seven time points for RNA extraction (see below). Erythromycin A was analysed by LCMS as described in [39].

### Total RNA isolation and mRNA enrichment

To protect RNA from endogenous RNA degradation, RNA isolation was performed after 8 h incubation at 4°C in RNA-later (Ambion). Total RNA was extracted using two cycles of cellular lysis in RNAse-free zirconia beads. RNA was purified using RNeasy midi kit (Qiagen) with on column DNase treatment and a second off column DNase treatment. Although the Qiagen columns have less binding affinity for RNAs smaller than 200 bp, it does not completely remove sRNAs [23] and are therefore suitable for sRNA analysis. RNA quality was evaluated using BioAnalyzer (Agilent) and Nanodrop prior analysis. Ribosomal RNA was removed with MicrobExpress Bacterial mRNA Enrichment kit (Ambion) or duplex-specific thermostable nuclease enzyme from Kamchatka crab (DSN) as described in the Illumina’s protocol. RNA samples quality and integrity was analyzed using 2100 BioAnalyzer (Agilent) according to manufacturer’s instructions.

### Microarrays and real-time quantitative PCR

Probes for microarrays, were designed using eArray and OligoRankPick [40] and evaluated using Agilent’s Base Composition (BC) algorithm. RNA was amplified using MessageAmp II-bacterial RNA Amplification kit (Life Technologies), reversed transcribed to cDNA, labeled using Agilent QuickAmp labeling kit (Life Technologies), hybridized, washed and scanned according to Agilent’s protocols. RT-PCR was performed from cDNA amplified from 600 ng of total RNA using Superscript III (Invitrogen) as per the manufacturer’s instructions for high GC% transcripts (50°C, 1 h). Diluted cDNA (1 in 50) was used for qRT PCRs performed in a Corbett Rotorgene 3000 using SYBRgreen UDG master-mix (Invitrogen). C_t_ values were determined using the default values on the Rotor Gene- 6 software. A correction for DNA contamination was performed using C_t_ values from RT^-^ controls. The concentration of cDNA (ng/reaction) for each sample was determined using corrected values. These values were then log_2_ transformed and normalized against *eryBV* (Additional file 1: Figure 1-i).

### Library preparation and sequencing

Deep sequencing was performed from libraries prepared as follow: Two libraries were prepared for total RNA, seven after DSN treatment [8] and the second round of sequencing (from an independent fermentation), following mRNA enrichment using MicrobExpress (Ambion), was sequenced strand specifically. Additionally, we ran a trial DSN-sequencing sample sequenced after preparation of three independent 90-nt, paired-end libraries. To achieve optimized clustering density, the trial run was sequenced with two 400mer libraries sequenced at two different concentrations following DSN normalization [41,42]. The third library was size selected from a gel after the final amplification to remove adapter multimer product and sequenced at 300mer. This optimized protocol for GC-rich microorganisms was used for subsequent DSN-sequencing. Libraries prepared after MicrobExpress mRNA enrichment were directionally sequenced at 90bp as described in the direction mRNA-seq prep pre-realised protocol from Illumina [43]. RNAs corresponding from 15-50nt fractions relative to ladder were excised for from the PAGE gel and purified for sequencing using the Illumina small RNA sequencing protocol with minor modifications as previously described [44]. All sequencing was performed at Geneworks (Adelaide, Australia) on the Illumina GAII. All Data is available at GEO GSE39722.

### Sequence read alignment and analysis

Reads were independently aligned to the *S.erythraea* genome using multiple software to assess for possible alignment bias caused by the high GC content of the genome. Reads were aligned using Burrows-Wheeler Aligner BWA [45] Bowtie [46] and Bowtie2 [47] requiring no more than 2 mismatches. Only reads that aligned to one genomic location were retained unless specified for the analysis. We employed CuffDiff [48] to determine gene expression using SAM files resulting from alignment, annotated gene file upper quantile normalization and masking of rRNA sequences. For expression of non-standard features, such as transcriptional macro-regions, read alignments were summed over genomic annotations. Hierarchal clustering of gene expression was performed by Pearson’s pair-wise complete linkage using Cluster3 [49]. Gene expression was normalized within libraries and between libraries as indicated in [50]. To determine significance of gene ontology enrichments, we performed Fisher’s exact test with multiple hypothesis correction. Additional statistical tests and figure generation were conducted using the Prism 5 (http://www.graphpad.com/prism/). Additional in-house perl scripts provided at http://matticklab.com/index.php?title=Marcel_Dinger was also employed. We also employed for figure generation Circos to generate circular genomic figures [51]. Operon prediction was performed from *de novo* assembly of all reads resulting from the strand specific biological replicate using Oases [17]. Reads were trimmed to 70 bases and assembled using a 45 bases kmer, from which 23% aligned with previously annotated operons (Additional file 2: Table S3, operon track in Gbrowser and *de novo* track in Gbrowser).

## Competing interests

The authors declare no competing financial interests.

## Authors’ contributions

TRM, EM and LKN designed the experiments. EM and CLC performed all fermentation and extractions. EM and CLC perform protein purification and analysis. TRM, RP and MED performed alignment, transcript assembly and analysis. JS performed RT-PCR, EM, TRM, CLC, JSM and LKN wrote the manuscript. All authors read and approved the final manuscript.

## Supplementary Material

Additional file 1 **Supplementary Data are available online.** All data is available on a dedicated *S. erythraea* genome browser (http://pathway.aibn.uq.edu.au/serythraea) and RNA-seq data has been submitted to GEO GSE39722 http://www.ncbi.nlm.nih.gov/geo/query/acc.cgi?token=pxcjzkcismecyze&acc=GSE39722 Click here for file

Additional file 2: Table S1 Summary of sequenced libraries and alignments. **Table S2.** Gene expression profiles for annotated genes. Gene identifier, description and normalised expression (RPKM) indicated. RPKM result from Cufflinks after removing multi-mappers (1), upper quantile normalization and masking of rRNA sequences. Bowtie2 alignments (2) are also presented. **Table S3.** Operon validated using Oases for de novo assemply of all reads. The majority of the DOOR operons present similarity with previous annotation (Mao et al., 2009). **Table S4.** Novel ncRNAs annotated with secondary strutture and dynamic profile across the feremntation. Unique identifier, chromosome location, and size indicated. **Table S5.** Gene-ontology analysis. Enrichment for GO terms at exponential, transitional and stationary phases within S.erythraea growth cycle. **Table S6.** Gene expression profiles for annotated genes. Gene identifier, description and expression before Quantile normalization (FPKM) indicated. Reads were aligned using Bowtie 2 and analysed using Cufflinks. Genes marked with "+" in Column J were considered highly expressed. Click here for file

## References

[B1] Bibb MJ Regulation of secondary metabolism in streptomycetes Curr Opin Microbiol 2005 8 2 208 215 15802254 10.1016/j.mib.2005.02.016

[B2] Oliynyk M Samborskyy M Lester JB Mironenko T Scott N Dickens S Haydock SF Leadlay PF Complete genome sequence of the erythromycin-producing bacterium Saccharopolyspora erythraea NRRL23338 Nat Biotechnol 2007 25 4 447 453 17369815 10.1038/nbt1297

[B3] Wu J Zhang Q Deng W Qian J Zhang S Liu W Toward improvement of erythromycin A production in an industrial Saccharopolyspora erythraea strain via facilitation of genetic manipulation with an artificial attB site for specific recombination Appl Environ Microbiol 2011 77 21 7508 7516 21841022 10.1128/AEM.06034-11 PMC3209160

[B4] Manteca A Sanchez J Jung HR Schwammle V Jensen ON Quantitative proteomics analysis of Streptomyces coelicolor development demonstrates that onset of secondary metabolism coincides with hypha differentiation Mol Cell Proteomics 2010 9 7 1423 1436 20224110 10.1074/mcp.M900449-MCP200 PMC2938082

[B5] Nieselt K Battke F Herbig A Bruheim P Wentzel A Jakobsen OM Sletta H Alam MT Merlo ME Moore J The dynamic architecture of the metabolic switch in Streptomyces coelicolor BMC Genomics 2010 11 10 20053288 10.1186/1471-2164-11-10 PMC2824715

[B6] Peano C Bicciato S Corti G Ferrari F Rizzi E Bonnal R Bordoni R Albertini A Bernardi L Donadio S Complete gene expression profiling of Saccharopolyspora erythraea using GeneChip DNA microarrays Microb Cell Fact 2007 6 1 37 18039355 10.1186/1475-2859-6-37 PMC2206050

[B7] Battke F Herbig A Wentzel A Jakobsen OM Bonin M Hodgson DA Wohlleben W Ellingsen TE Nieselt K A technical platform for generating reproducible expression data from Streptomyces coelicolor batch cultivations Adv Exp Med Biol 2011 696 3 15 21431541 10.1007/978-1-4419-7046-6_1

[B8] Zhulidov PA Bogdanova EA Shcheglov AS Vagner LL Khaspekov GL Kozhemyako VB Matz MV Meleshkevitch E Moroz LL Lukyanov SA Simple cDNA normalization using kamchatka crab duplex-specific nuclease Nucleic Acids Res 2004 32 3 e37 14973331 10.1093/nar/gnh031 PMC373426

[B9] He S Wurtzel O Singh K Froula JL Yilmaz S Tringe SG Wang Z Chen F Lindquist EA Sorek R Validation of two ribosomal RNA removal methods for microbial metatranscriptomics Nat Meth 2010 7 10 807 812 10.1038/nmeth.1507 20852648

[B10] Shagina I Bogdanova E Mamedov IZ Lebedev Y Lukyanov S Shagin D Normalization of genomic DNA using duplex-specific nuclease Biotechniques 2010 48 6 455 459 20569220 10.2144/000113422

[B11] Kapranov P Cheng J Dike S Nix DA Duttagupta R Willingham AT Stadler PF Hertel J Hackermuller J Hofacker IL RNA Maps Reveal New RNA Classes and a Possible Function for Pervasive Transcription Science 2007 316 5830 1484 1488 17510325 10.1126/science.1138341

[B12] Nagalakshmi U Wang Z Waern K Shou C Raha D Gerstein M Snyder M The Transcriptional Landscape of the Yeast Genome Defined by RNA Sequencing Science 2008 320 5881 1344 1349 18451266 10.1126/science.1158441 PMC2951732

[B13] Brewer BJ When polymerases collide: Replication and the transcriptional organization of the E. coli chromosome Cell 1988 53 5 679 686 3286014 10.1016/0092-8674(88)90086-4

[B14] Hopwood DA Soil to genomics: the Streptomyces chromosome Annu Rev Genet 2006 40 1 23 16761950 10.1146/annurev.genet.40.110405.090639

[B15] Martin JF Santos-Beneit F Rodriguez-Garcia A Sola-Landa A Smith MC Ellingsen TE Nieselt K Burroughs NJ Wellington EM Transcriptomic studies of phosphate control of primary and secondary metabolism in Streptomyces coelicolor Appl Microbiol Biotechnol 2012 95 1 61 75 22622839 10.1007/s00253-012-4129-6

[B16] Mao F Dam P Chou J Olman V Xu Y DOOR: a database for prokaryotic operons Nucleic Acids Res 2009 37 suppl 1 D459 D463 18988623 10.1093/nar/gkn757 PMC2686520

[B17] Schulz MH Zerbino DR Vingron M Birney E Oases: robust de novo RNA-seq assembly across the dynamic range of expression levels Bioinformatics 2012 28 8 1086 1092 22368243 10.1093/bioinformatics/bts094 PMC3324515

[B18] Kong L Zhang Y Ye Z-Q Liu X-Q Zhao S-Q Wei L Gao G CPC: assess the protein-coding potential of transcripts using sequence features and support vector machine Nucleic Acids Res 2007 35 suppl 2 W345 W349 17631615 10.1093/nar/gkm391 PMC1933232

[B19] Passalacqua KD Varadarajan A Ondov BD Okou DT Zwick ME Bergman NH The Structure and Complexity of a Bacterial Transcriptome J Bacteriol 2009 191 10 3203 3211 19304856 10.1128/JB.00122-09 PMC2687165

[B20] Manteca A Fernandez M Sanchez J Cytological and biochemical evidence for an early cell dismantling event in surface cultures of Streptomyces antibioticus Res Microbiol 2006 157 2 143 152 16171979 10.1016/j.resmic.2005.07.003

[B21] Gatewood ML Bralley P Weil MR Jones GH The transcriptome of Streptomyces coelicolor: RNA-seq and RNA immunoprecipitation identify substrates for RNase III J Bacteriol 2012 Published ahead of print 10.1128/JB.06541-11 PMC3347082 22389483

[B22] Jones GH RNA degradation and the regulation of antibiotic synthesis in Streptomyces Future Microbiol 2010 5 3 419 429 20210552 10.2217/fmb.10.14

[B23] Qiu Y Cho BK Park YS Lovley D Palsson BO Zengler K Structural and operational complexity of the Geobacter sulfurreducens genome Genome Res 2010 20 9 1304 1311 20592237 10.1101/gr.107540.110 PMC2928509

[B24] Hatoum-Aslan A Maniv I Marraffini LA Mature clustered, regularly interspaced, short palindromic repeats RNA (crRNA) length is measured by a ruler mechanism anchored at the precursor processing site Proc Natl Acad Sci U S A 2011 108 52 21218 21222 22160698 10.1073/pnas.1112832108 PMC3248500

[B25] Sharma CM Hoffmann S Darfeuille F Reignier J Findeisz S Sittka A Chabas S Reiche K Hackermuller J Reinhardt R The primary transcriptome of the major human pathogen Helicobacter pylori Nature 2010 464 7286 250 255 20164839 10.1038/nature08756

[B26] McCormick JR Flärdh K Signals and regulators that govern Streptomyces development FEMS Microbiol Rev 2012 36 1 22092088 10.1111/j.1574-6976.2011.00317.x PMC3285474

[B27] Hindra Pak P Elliot MA Regulation of a novel gene cluster involved in secondary metabolite production in Streptomyces coelicolor J Bacteriol 2010 192 19 4973 4982 20675485 10.1128/JB.00681-10 PMC2944504

[B28] Xu W Huang J Lin R Shi J Cohen SN Regulation of morphological differentiation in S. coelicolor by RNase III (AbsB) cleavage of mRNA encoding the AdpA transcription factor Mol Microbiol 2010 75 3 781 791 20059679 10.1111/j.1365-2958.2009.07023.x PMC2936110

[B29] Bentley SD Chater KF Cerdeno-Tarraga AM Challis GL Thomson NR James KD Harris DE Quail MA Kieser H Harper D Complete genome sequence of the model actinomycete Streptomyces coelicolor A3(2) Nature 2002 417 6885 141 147 12000953 10.1038/417141a

[B30] Choulet F Gallois A Aigle B Mangenot S Gerbaud C Truong C Francou F-X Borges F Fourrier C Guerineau M Intraspecific Variability of the Terminal Inverted Repeats of the Linear Chromosome of Streptomyces ambofaciens J Bacteriol 2006 188 18 6599 6610 16952952 10.1128/JB.00734-06 PMC1595491

[B31] Umbarger MA Toro E Wright MA Porreca GJ Bau D Hong SH Fero MJ Zhu LJ Marti-Renom MA McAdams HH The three-dimensional architecture of a bacterial genome and its alteration by genetic perturbation Mol Cell 2011 44 2 252 264 22017872 10.1016/j.molcel.2011.09.010 PMC3874842

[B32] Harigaya Y Parker R Global analysis of mRNA decay intermediates in Saccharomyces cerevisiae Proc Natl Acad Sci U S A 2012 109 29 11764 11769 22752303 10.1073/pnas.1119741109 PMC3406813

[B33] Miller C Schwalb B Maier K Schulz D Dumcke S Zacher B Mayer A Sydow J Marcinowski L Dolken L Dynamic transcriptome analysis measures rates of mRNA synthesis and decay in yeast Mol Syst Biol 2011 7 458 21206491 10.1038/msb.2010.112 PMC3049410

[B34] Gravenbeek ML Jones GH The endonuclease activity of RNase III is required for the regulation of antibiotic production by Streptomyces coelicolor Microbiology 2008 154 Pt 11 3547 3555 18957607 10.1099/mic.0.2008/022095-0

[B35] Dalebroux ZD Swanson MS ppGpp magic beyond RNA polymerase Nat Rev Microbiol 2012 10 3 203 212 22337166 10.1038/nrmicro2720 PMC13198741

[B36] Gatewood ML Jones GH (p)ppGpp inhibits polynucleotide phosphorylase from streptomyces but not from Escherichia coli and increases the stability of bulk mRNA in Streptomyces coelicolor J Bacteriol 2010 192 17 4275 4280 20581211 10.1128/JB.00367-10 PMC2937373

[B37] Siculella L Damiano F di Summa R Tredici SM Alduina R Gnoni GV Alifano P Guanosine 5'-diphosphate 3'-diphosphate (ppGpp) as a negative modulator of polynucleotide phosphorylase activity in a 'rare' actinomycete Mol Microbiol 2010 77 3 716 729 20545843 10.1111/j.1365-2958.2010.07240.x

[B38] Carata E Peano C Tredici SM Ferrari F Tala A Corti G Bicciato S De Bellis G Alifano P Phenotypes and gene expression profiles of Saccharopolyspora erythraea rifampicin-resistant (rif) mutants affected in erythromycin production Microb Cell Fact 2009 8 18 19331655 10.1186/1475-2859-8-18 PMC2667423

[B39] Licona-Cassani C Marcellin E Quek LE Jacob S Nielsen LK Reconstruction of the Saccharopolyspora erythraea genome-scale model and its use for enhancing erythromycin production Antonie Van Leeuwenhoek 2012 102 3 493 502 22847261 10.1007/s10482-012-9783-2

[B40] Hu G Llinas M Li J Preiser PR Bozdech Z Selection of long oligonucleotides for gene expression microarrays using weighted rank-sum strategy BMC Bioinformatics 2007 8 350 17880708 10.1186/1471-2105-8-350 PMC2099447

[B41] DSN Normalization Sample Prep Guide Pilot Release

[B42] Storz G Vogel J Wassarman Karen M Regulation by Small RNAs in Bacteria: Expanding Frontiers Mol Cell 2011 43 6 880 891 21925377 10.1016/j.molcel.2011.08.022 PMC3176440

[B43] Directional mRNA-Seq Library Prep Pre-Release Protocol

[B44] Taft RJ Simons C Nahkuri S Oey H Korbie DJ Mercer TR Holst J Ritchie W Wong JJ Rasko JE Nuclear-localized tiny RNAs are associated with transcription initiation and splice sites in metazoans Nat Struct Mol Biol 2010 17 8 1030 1034 20622877 10.1038/nsmb.1841

[B45] Li H Durbin R Fast and accurate short read alignment with Burrows-Wheeler transform Bioinformatics 2009 25 14 1754 1760 19451168 10.1093/bioinformatics/btp324 PMC2705234

[B46] Langmead B Trapnell C Pop M Salzberg S Ultrafast and memory-efficient alignment of short DNA sequences to the human genome Genome Biol 2009 10 3 R25 19261174 10.1186/gb-2009-10-3-r25 PMC2690996

[B47] Langmead B Salzberg SL Fast gapped-read alignment with Bowtie 2 Nat Methods 2012 9 4 357 359 22388286 10.1038/nmeth.1923 PMC3322381

[B48] Trapnell C Williams BA Pertea G Mortazavi A Kwan G van Baren MJ Salzberg SL Wold BJ Pachter L Transcript assembly and quantification by RNA-Seq reveals unannotated transcripts and isoform switching during cell differentiation Nat Biotech 2010 28 5 511 515 10.1038/nbt.1621 PMC3146043 20436464

[B49] de Hoon MJL Imoto S Nolan J Miyano S Open source clustering software Bioinformatics 2004 20 9 1453 1454 14871861 10.1093/bioinformatics/bth078

[B50] Bullard JH Purdom E Hansen KD Dudoit S Evaluation of statistical methods for normalization and differential expression in mRNA-Seq experiments BMC Bioinformatics 2010 11 94 20167110 10.1186/1471-2105-11-94 PMC2838869

[B51] Krzywinski M Schein J Birol I Connors J Gascoyne R Horsman D Jones SJ Marra MA Circos: An information aesthetic for comparative genomics Genome Res 2009 19 9 1639 1645 19541911 10.1101/gr.092759.109 PMC2752132

