# Supplementary material for: Saccharopolyspora erythraea’s genome is organised in high-order transcriptional regions mediated by targeted degradation at the metabolic switch
Source: BMC Genomics. 2013 Jan 16;14:15. doi: 10.1186/1471-2164-14-15 (PMC3610266; doi:10.1186/1471-2164-14-15)
Supplement: Additional file 1 — Supplementary Data are available online. All data is available on a dedicated S. erythraea genome browser (http://pathway.aibn.uq.edu.au/serythraea) and RNA-seq data has been submitted to GEO GSE39722 http://www.ncbi.nlm.nih.gov/geo/query/acc.cgi?token=pxcjzkcismecyze&acc=GSE39722 [file 1471-2164-14-15-S1.docx]

***Saccharopolyspora erythraea’s* genome is organised in high-order transcriptional regions mediated by targeted degradation at the metabolic switch**

**Esteban Marcellin^1,3^, Tim R. Mercer^2,3^, Cuauhtemoc Licona-Cassani^1^, Robin W. Palfreyman**^1^**, Marcel E. Dinger^2^, Jennifer A. Steen^1^, John S. Mattick^2^ and Lars K. Nielsen^1,4^**

^1^Australian Institute for Bioengineering and Nanotechnology (AIBN), The University of Queensland, Qld 4072, Australia

^2^Institute for Molecular Bioscience (IMB), The University of Queensland, Brisbane, QLD 4067, Australia.

^3^ Authors contributed equally to this manuscript.

^4^To whom correspondence should be addressed:

Lars K. Nielsen

Telephone: + 61 7 334 63986

Fax: + 61 7 334 63973

Email: lars.nielsen@uq.edu.au

**SUPPLEMENTARY INFORMATION:**

1. FIGURES (page 2)

2. TABLE LEGENDS (page 9)

**Supplementary Figure S1. (A)** Genome schematic showing distribution of genes for which microarray probes can be satisfactorily scored using the Agilent eArray Probe Design Algorithm (satisfactory probes indicated in green; suboptimal in yellow, unable to be included within design indicated in red, GC content black). (**B)** Relative nucleotide fractions of sequenced reads from DSN-, MicrobExpress-treated and untreated libraries with genome and annotated genes nucleotide composition included for comparison. **(C)** Fractional nucleotide composition of genes enriched (green) or depleted (blue) in DSN-treated relative to untreated libraries. **(D)** Cumulative frequency distribution showing the fractional G+C composition of transcripts enriched (green) or depleted (blue) in DSN and MicrobExpress-treated relative to untreated library. **(E)** Comparative scatter-plot indicating no correlation (*r^2^*=0.01) between gene expression and fractional G+C content in DSN-treated libraries. **(F)** Cumulative frequency distribution indicating population structure of gene abundance in untreated (red) and MicrobExpress- (green) and DSN-normalised (blue) libraries. **(g-h)** Scatter-plot comparison of gene expression estimates between untreated and MicrobExpress- **(G)** and DSN-normalised **(H)** libraries. **(I)** Validation of RNA sequencing gene abundance estimates (blue) by qRT-PCR (purple). Values were log2 transformed and normalised against *eryBV*. **(J)** Scatterplot comparison of relative changes in gene expression as determined by microarray (for satisfactory probes) and matching RNA sequencing. Values were then log2 transformed and normalised against *eryBV*.

**Supplementary Figure S2. (A)** Hierarchal clustering (Pearson’s) of repetitive transposases encoded within the *S.erythraea* genome. A select subset (green) that are induced during the switch and localised to the core/noncore boundaries are indicated. **(B)** Cumulative frequency distribution of maximal gene fold change. **(C)** Profile of replicate *S.erythraea* fermentation cycle according to cell density and erythromycin production (grey). Primary (red), transition (purple) and secondary (blue) metabolic phases are indicated.

**Supplementary Figure S3. (A)** Hierarchal clustering (Pearson’s) of genes associated with secondary metabolism. Erythrmoycin cluster indicated. **(B)** Expression profile of genes within Erythromycin cluster in order of genome. **(C)** Genome browser view showing strand–specific read alignment across detail from erythromycin gene cluster.

**
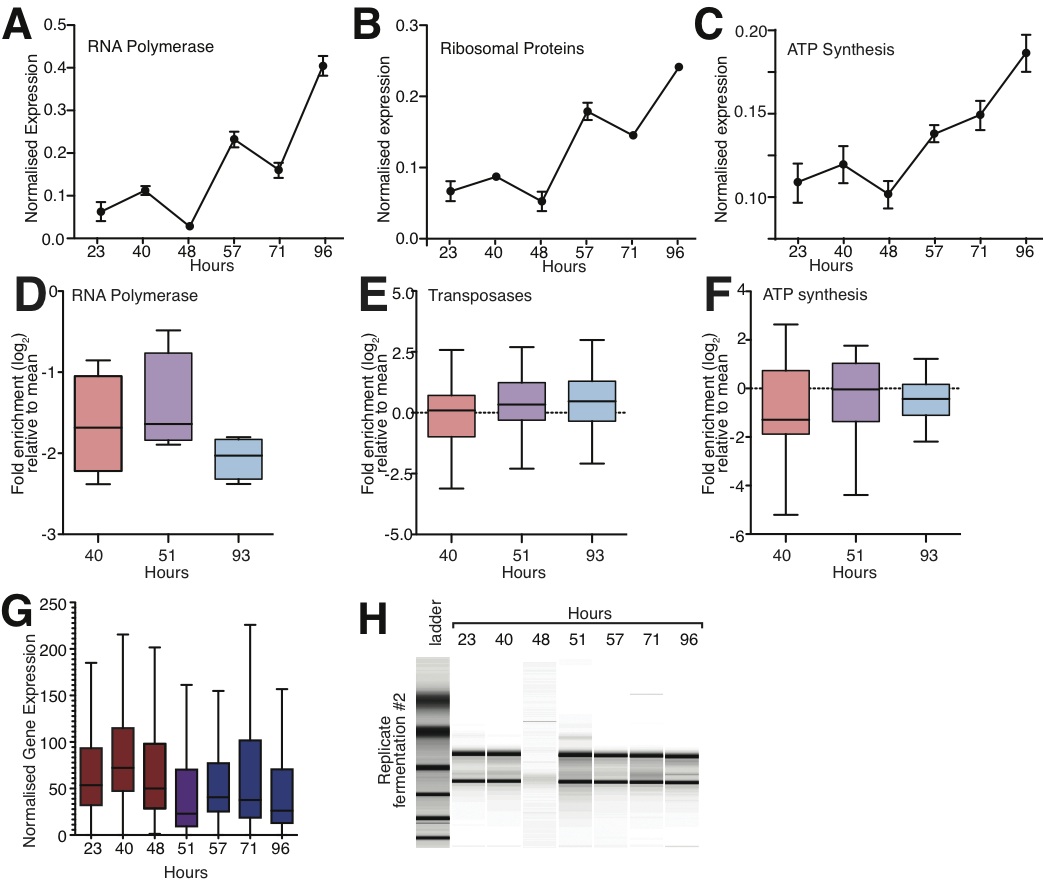
Supplementary Figure S4. (A-C)** Normalised mean expression profile (bars indicate standard deviation) core components of RNA polymerase **(A)** Ribosomal proteins **(B)** and ATP synthesis **(C)** across *S.erythraea* life cycle. Sample 51 was omitted. **(D,E,F)** Box-whisker plot (Tukey) showing the relative fold-enrichment of RNA fragments to full length RNAs for genes associated with RNA polymerase **(D),** transposases **(E)** and ATP synthesis **(F). (G)** Box-whisker plot (Tukey distribution) showing the normalised expression of genes (per kb per million of total mRNA). **(H)** Gel electrophoresis of sampled RNA from *S.erythraea* replicate fermentation showing specific RNA degradation at switch (in the second fermentation happening at 48 h) (BioAnalyzer tracer).

**
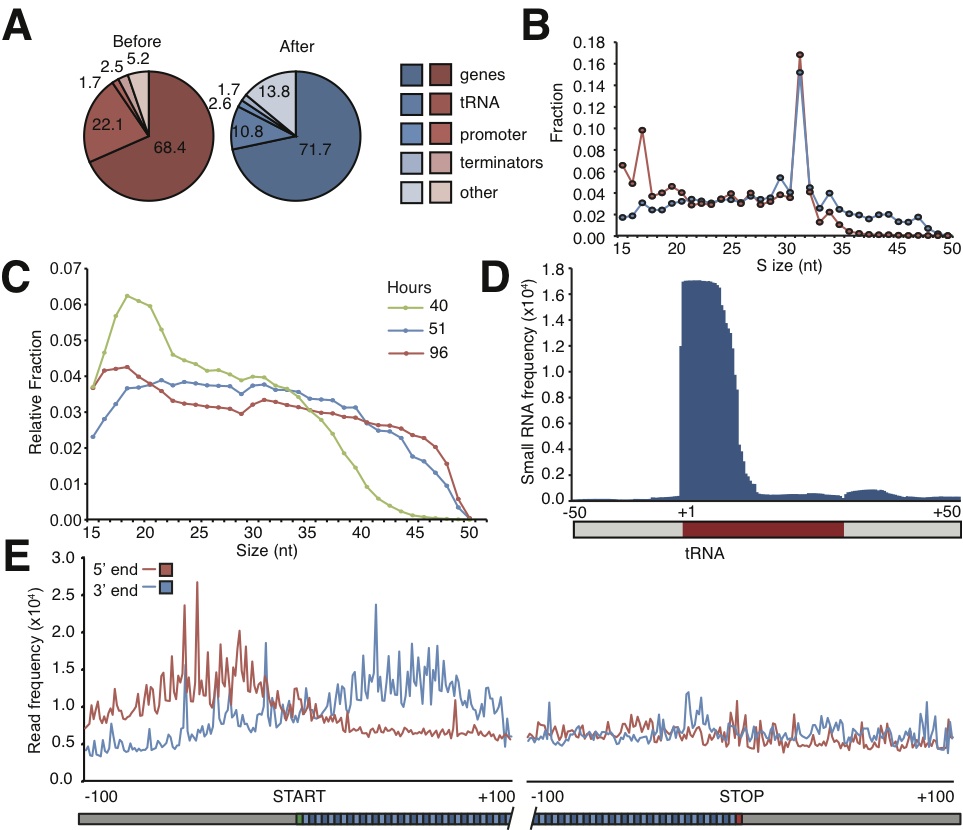
Supplementary Figure S5. (A)** Pie graphs indicating the proportion of sequenced small (15-50nt) RNA fragments aligning to features of the *S.erythraea* genome. **(B,C)** Size distribution of total small RNA sequenced reads **(B)** and sequenced reads aligning sense to annotated genes **(C).** Total reads exhibit distinct enrichment for 32nt reads, the majority of which align to tRNA. **(D)** Cumulative alignment across tRNA loci shows sensitivity to process tRNAs that contain 5’ monophosphate, as opposed to primary tRNA transcript containing 3’ triphosphate. **(E)** Frequency distribution of full length RNA 5’ (red) and 3’ (blue) termini aligning sense to mRNA strand shows no underlying sequence bias to alignment frequency.

**
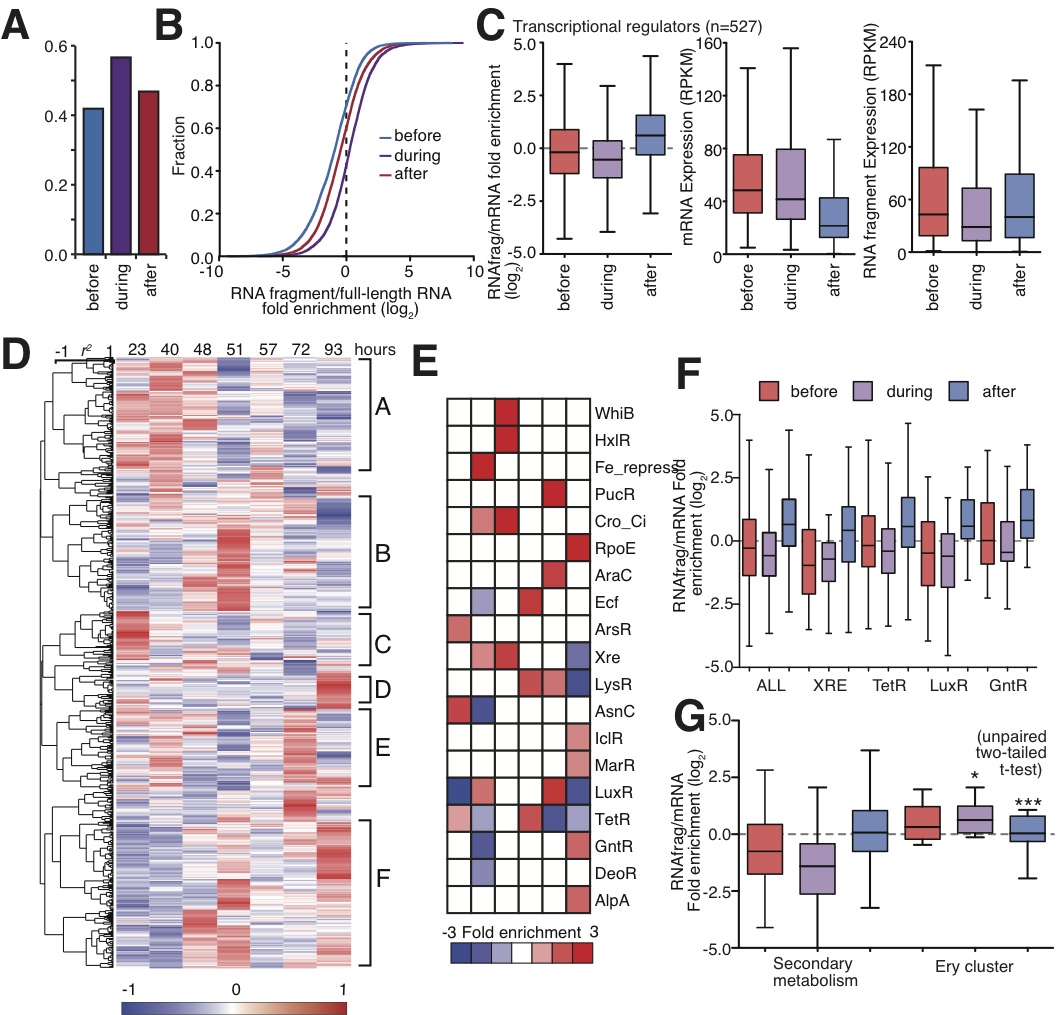
Supplementary Figure S6. (A)** Bar-graph indicates the relative proportion of small RNA sequenced libraries that align in sense direction to annotated genes. **(B)** Cumulative frequency distribution of fold enrichment of RNA fragments to full length RNAs for annotated genes indicates that during the switch (purple) we observed elevated degradation of mRNAs relative to other stages. **(C)** Box-whisker plot (Tukey distribution) showing the fold enrichment of RNA fragments to full-length RNA before indicates accelerated degradation following the switch. **(B,C)** Box-whisker plot showing the full length **(D)** and RNA fragment **(C)** alignment frequency for annotated transcriptional regulators. (**D)** Hierarchal clustering (Pearson’s) of transcription factor regulators illustrates clusters of co-expressed genes. **(E)** Grid plot indicates the significant enrichment for transcriptional factor classes in clusters identified from hierarchal clustering. **(F)** Box-whisker plot indicates fold enrichment for RNA fragments to full-length RNA for specific transcription factor families before (red), during (purple) and after (blue) the switch. **(G)** Box-whisker plot indicates the fold enrichment for RNA fragments to full-length RNA for genes associated with secondary metabolism and the erythromycin cluster.

**
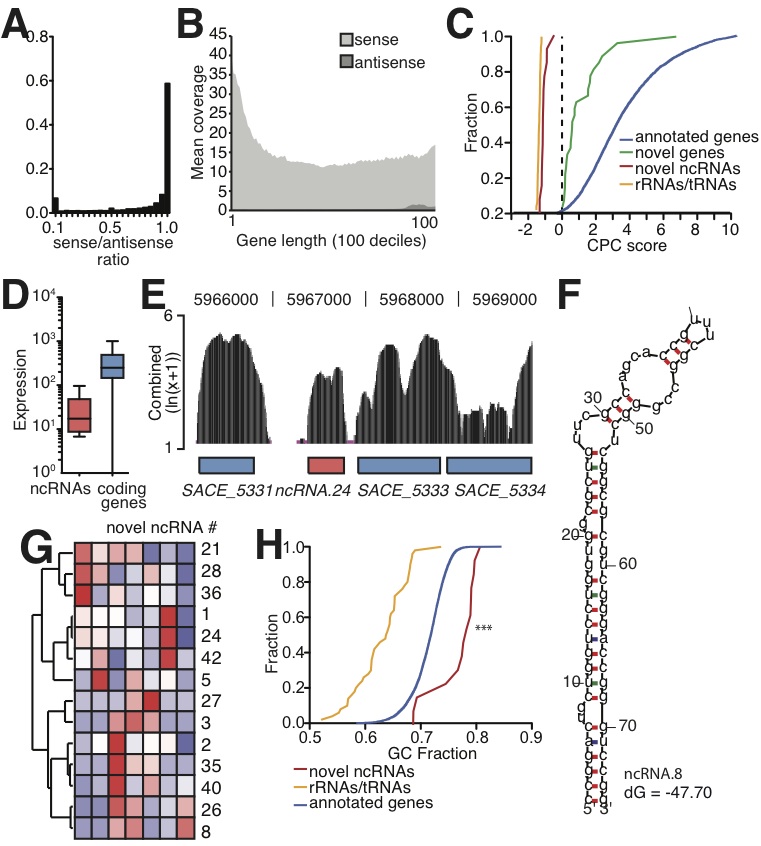
**

**Supplementary Figure S7.** Functional ncRNA prediction and annotation **(A and B)** Cumulative distribution of aligned reads in sense (light gray) and antisense (dark gray) across gene body. **(C)** Cumulative frequency distribution of Coding Predictor Calculator (CPC) for identified ncRNAs (red). Protein-coding genes (blue) and tRNAs/rRNAs (yellow) included for comparison/ **(D)** Box-whisker plot (Tukey distribution) showing ncRNA (red) expression relative to annotated protein-coding genes (blue). **(E)** Genome browser view showing alignment distribution associated with identified ncRNA (red box) and adjacent protein-coding genes (blue box). **(F)** Predicted RNA secondary structure within identified ncRNA. **(G)** Normalised expression profiles of 14 identified ncRNAs. **(H)** Cumulative frequency distribution showing fraction G+C composition of ncRNAs (red) relative to coding genes (blue) and rRNAs/tRNAs (yellow).

**
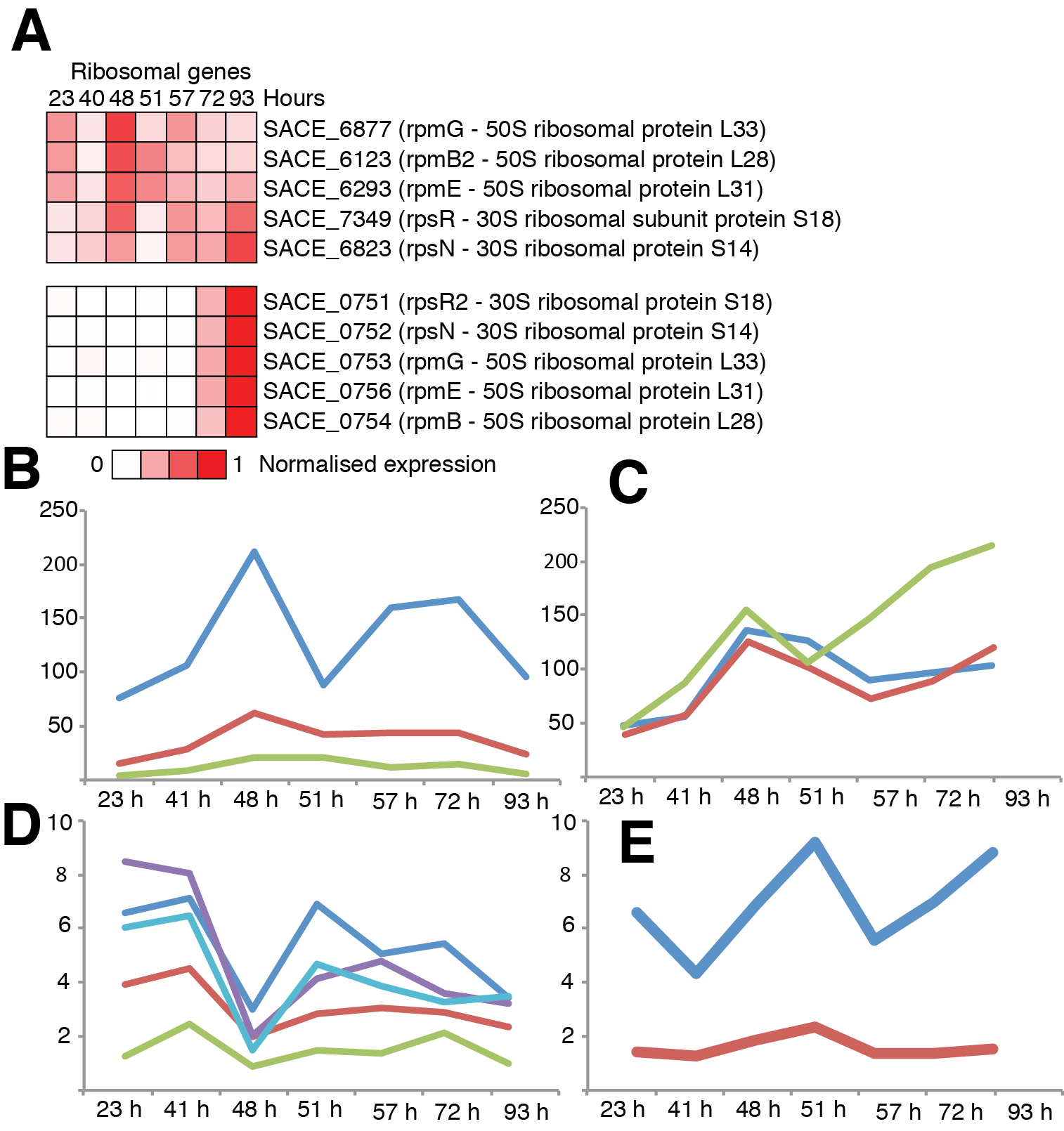
**

**Supplementary Figure S8. (A)** Expression profile of selected ribosomal protein genes (upper panel) exhibits discordant expression to paralogous operon (middle panel). **(B)** Expression profile of SACE_4683 (blue), SACE_4684 (red) and SACE_4685 (green) after normalization with libraries. **(C)** Expression profile of SACE_0411 (blue), SACE_0412 (red) and SACE_0413 (green) after normalization with libraries. **(D)** Expression profile of SACE_4683 (blue), SACE_4684 (red) and SACE_4685 (green) after normalization with libraries. **(E)** Expression profile of SACE_6085 -SACE_6087 (Blue, red, green, light blue, purple) after normalization with libraries.

**SUPPLEMENTARY TABLES**

**Supplementary Table 1. Summary of sequenced libraries and alignments**

**Supplementary Table 2. Gene expression profiles for annotated genes.** Gene identifier, description and normalised expression (RPKM) indicated. RPKM result from Cufflinks after removing multi-mappers (1), upper quantile normalization and masking of rRNA sequences. Bowtie2 alignments (2) are also presented.

**Supplementary Table 3**. **Operon validated using Oases for de novo assemply of all reads**. The majority of the DOOR operons present similarity with previous annotation (Mao et al., 2009)

**Supplementary Table 4. Novel ncRNAs annotated.** Unique identifier, chromosome location, and size indicated.

**Supplementary Table 5. Gene-ontology analysis.** Enrichment for GO terms at exponential, transitional and stationary phases within *S.erythraea* growth cycle.

**Supplementary Table S6. Gene expression profiles for annotated genes.** Gene identifier, description and expression before Quantile normalization (FPKM) indicated.

Reads were aligned using Bowtie 2 and analysed using Cufflinks. Genes marked with "+" in Column J were considered highly expressed.
